# Frailty reduces penumbral volumes and attenuates treatment response in hyperacute ischemic stroke

**DOI:** 10.1093/ageing/afae266

**Published:** 2024-12-01

**Authors:** Esmee Dohle, Benjamin Lewis, Smriti Agarwal, Elizabeth A Warburton, Nicholas R Evans

**Affiliations:** Department of Clinical Neurosciences, University of Cambridge, R3 Clinical Neurosciences (Box 83) Addenbrooke's Hospital Hills Road, Cambridge CB2 0QQ, UK; Department of Clinical Neurosciences, University of Cambridge, R3 Clinical Neurosciences (Box 83) Addenbrooke's Hospital Hills Road, Cambridge CB2 0QQ, UK; Department of Clinical Neurosciences, University of Cambridge, R3 Clinical Neurosciences (Box 83) Addenbrooke's Hospital Hills Road, Cambridge CB2 0QQ, UK; Department of Clinical Neurosciences, University of Cambridge, R3 Clinical Neurosciences (Box 83) Addenbrooke's Hospital Hills Road, Cambridge CB2 0QQ, UK; Department of Clinical Neurosciences, University of Cambridge, R3 Clinical Neurosciences (Box 83) Addenbrooke's Hospital Hills Road, Cambridge CB2 0QQ, UK

**Keywords:** frailty, strokes, penumbral, ischemic, thrombolysis, older people

## Abstract

**Background:**

Frailty—the loss of physiological reserve to withstand a stressor event—is associated with poorer outcomes following acute stroke reperfusion therapies. However, the mechanisms underlying this relationship are poorly understood. This study investigated the association between frailty and penumbral volumes in hyperacute ischemic stroke.

**Methods:**

Total ischemic lesion volumes (comprising infarct core and penumbral volumes) were measured using computed tomography (CT) perfusion imaging to give the penumbral fraction within the ischemic lesion. Pre-stroke frailty was measured using a validated frailty index. The relationship between frailty and penumbral fraction was adjusted for age, onset-to-CT interval, collateral scores, small vessel disease burden and vascular comorbidities. Stroke severity was measured using the National Institutes of Health Stroke Scale at baseline and after 24 h.

**Results:**

In 55 individuals receiving thrombolysis for ischemic stroke, increasing frailty was associated with a reduction in penumbral fraction (*r_s_* = −0.36, *P* < 0.01). This remained significant after adjustment for age, onset-to-imaging time and collateral score (beta = −1.16, *P* < 0.001). Correspondingly, frailty was independently negatively associated with proportional improvement in stroke severity following treatment (beta = −2.00, *P* < 0.01). C-reactive protein (CRP) on presentation was associated with frailty index (*r_s_* = 0.38, *P* < 0.01) and penumbral fraction (*r_s_* = −0.30, *P* = 0.02).

**Discussion:**

A reduction in salvageable penumbra in frailty may explain the treatment-attenuating effects of frailty on reperfusion therapies. The association with CRP motivates further research into a possible inflammatory component of this relationship.

**Conclusion:**

Frailty is independently associated with reduced penumbra and poorer neurological recovery in acute stroke. These findings may explain the attenuated response to stroke reperfusion therapies seen in frailer individuals.

## Key Points

- Frailty is associated with a reduction in salvageable penumbra in stroke.
- Reduced penumbral fraction seen in frailty may explain the attenuated response to stroke reperfusion therapies.
- Frailty is independently associated with attenuated neurological recovery in acute ischemic stroke.

## Introduction

Frailty is a clinical syndrome characterised by a reduced ability to maintain homeostasis in response to acute stressors, typified by decreasing physiological reserve [1, 2]. Although frequently concurrent with age and disability, frailty is a distinct syndrome that is an independent risk factor for poor outcomes across a range of medical conditions [1]. Frailty is common amongst individuals presenting with stroke, with a degree of pre-stroke frailty found in two-thirds of stroke patients [3]. Frailty is increasingly recognised to have both disease- and treatment-modifying effects across all stages of stroke presentation and recovery, and may serve as a stronger predictor of outcome than the pre-morbid modified Rankin score (mRS) [4, 5]. In the acute setting, pre-stroke frailty is associated with poorer recovery and increased mortality after ischemic stroke [6], whilst pre-stroke frailty appears to attenuate the benefit from reperfusion therapies [6, 7]. However, the mechanisms underlying this treatment-modifying effect remain unclear.

Given the complex natures of both frailty and the ischemic penumbra, there are a number of possible shared pathways that may be implicated. Associations between frailty and systemic inflammation have been reported [8], whilst systemic inflammation has been implicated in blood–brain barrier disruption [9] and chronic cerebrovascular disease [10]. The collateral circulation has been found to become impaired with age, [11] though the association between collateral supply and frailty has not yet been described. However, frailty has been reported to be associated with a number of other structural brain changes, including atrophy and leukoaraiosis [12, 13]. Finally, it has been postulated that cerebral physiology may change with age independently of any structural effects on the brain or collateral supply [14].

This study aims to establish the relationship between pre-stroke frailty, the extent of salvageable penumbra and consequent early neurological change in hyperacute ischemic stroke cases receiving reperfusion therapy. As a secondary outcome, we evaluated the relationship between frailty and salvageable penumbra with routinely collected markers of inflammation. Given the previously described associations between frailty and attenuated neurological recovery after reperfusion therapies, we hypothesised that frailty was associated with reduced salvageable penumbra in hyperacute ischemic stroke.

## Methods

### Study cohort

All individuals presenting to our centre within 4.5 h of ischemic stroke onset between 1 January 2019 and 31 December 2019 routinely underwent computed tomography (CT), CT angiography (CTA) and CT perfusion (CTP). Only individuals with a confirmed perfusion abnormality corresponding to the acute neurological deficit were included in this study.

Electronic medical records were retrospectively reviewed to extract age, sex, vascular comorbidities (hypertension, atrial fibrillation, smoking and diabetes mellitus), time from symptom-onset to CT, National Institutes of Health Stroke Scale (NIHSS) at presentation and after 24 h and pre-morbid modified Rankin Scale (mRS).

### Frailty measurement

Frailty was quantified using a frailty index (FI) used previously to assess frailty in individuals with stroke, where 33 characteristics (‘deficits’) are assessed across domains encompassing physical function, domicile status, comorbidities and biochemical results to yield a FI between 0 and 1 (Table 1). [15–17] FI was measured based on the individual’s status two weeks prior to the stroke, and was blinded to imaging readings and clinical outcomes (ED and BL). For dichotomized analyses, a FI cut-off of ≥0.24 was used between frailty and non-frailty. [18]

**Table 1:** Constituent deficits of the frailty index.

Anxiety	Haemoglobin (low)	Mobility aid
Depression	Care home resident	Assistance walking
Polypharmacy	Carers	Hypo/hypercalcaemia
Atrial fibrillation	Sensory impairment	Albumin (low)
Diabetes mellitus	Hearing aid	Hyperglycaemia
Hypertension	Bladder incontinence	Renal failure
Heart failure	Bowel incontinence	Liver disease
Vascular disease	Falls	Peptic ulcer
Hyperlipidaemia	Previous fracture	Arthritis
Previous myocardial infarction	Impaired activities of daily living	Cancer
Previous cerebrovascular disease	Impaired external activities of daily living	Chronic obstructive pulmonary disease

### Penumbral measurement

Cerebral perfusion volumes were automatically calculated using MIStar software (Apollo Medical Imaging Technology, Melbourne, Australia). Detection thresholds measured the volume (mL) of ischemia [cerebral blood flow (CBF) <30% relative to normal brain tissue] and infarct core (time-to-maximum >3.0 s). Penumbral fraction was defined as the difference between these volumes divided by the total volume of ischemia, yielding a score of 0–1. CTP images were visually assessed to ensure that the regions identified as penumbra and core were located in the appropriate neurovascular territory corresponding to the participant’s symptoms, and that there were no other areas of artefact affecting the volume measurements.

### Assessment of collateral circulation

Collateral status was evaluated using validated methodology, where reconstructed axial CTA were assigned collateral scores by an experienced neurovascular researcher (SA) based on vessels at the Sylvian fissure and the leptomeningeal convexity [19].

### Assessment of small vessel disease

The severity of small vessel disease was measured using the Fazeka scale adapted for use in CT imaging, generating a severity score between 0 and 3 [20]. This was scored by an experienced neurovascular researcher (NRE) from the baseline non-contrast CT head using the contralateral hemisphere to the acute ischemic lesion, blinded to clinical details and outcome measures.

### Inflammatory markers

Blood tests were taken on arrival in hospital to measure serum C-reactive protein (CRP) and neutrophil:lymphocyte ratio (NLR). Results were available for all participants, and no individual had overt clinical, biochemical or radiological signs of concurrent infection at the time of presentation.

### Statistical analysis

In univariable analysis, continuous data were tested for normality using Shapiro–Wilk testing and compared using parametric or non-parametric testing as appropriate. Proportions of categorical variables were compared using the two proportion Z-test. Linear regression adjusted for age, sex, onset-to-imaging interval, collateral score and vascular comorbidities (hypertension, atrial fibrillation, smoking and diabetes mellitus), with model refinement using stepwise regression using backwards elimination determined by the Akaike information criterion method [21], a widely used simple and versatile method of model selection that is asymptotically consistent and penalises over-parameterization [22]. Assumptions for linear regression modelling (linearity, independence, homoscedasticity and normality of residuals) were tested through Durbin–Watson testing and visualisation of residual plots and fulfilled. Mediation analysis was conducted using Sobel testing.

### Standard protocol approvals, registrations and patient consents

The project received local Institutional Review Board approval (Safety and Quality Support Department, Cambridge University Hospitals NHS Foundation Trust, project PRN11693). As the project was a retrospective analysis of investigations undertaken as part of routine clinical care, the need for participant consent was waived.

### Data availability

The corresponding author had full access to all the data in the study and takes responsibility for its integrity and the data analysis. The data supporting the findings of this study are available from the corresponding author upon reasonable request. Reporting followed STROBE guidelines.

## Results

### Baseline characteristics and aetiology

A total of 55 individuals presented during the study period with an acute perfusion deficit on CTP. Baseline participant characteristics are shown in Table 2.

**Table 2:** Baseline characteristics.

Median age (years) (IQR).	75 (63–84)
Male patients (%)	27 (49.1)
Median onset-to-CT interval (minutes) (IQR)	135 (99.0–195.5)
Median presentation NIHSS (IQR)	11 (5.5–19)
Current/former smoker (%)	26 (47.3)
Hypertension (%)	32 (58.2)
Diabetes (%)	4 (7.3)
Atrial fibrillation (%)	18 (32.7)

Using a dichotomized approach to frailty, 14 (25.5%) individuals were frail, and 41 (74.5%) were non-frail.

Age and FI showed only a moderate association on univariable analysis (*r_s_* = 0.54, *P* < 0.001). FI and mRS also showed only a moderate association on univariable analysis (*r_s_* = 0.59, *P* < 0.001).

### FI and cerebral perfusion parameters

**Figure 1:**
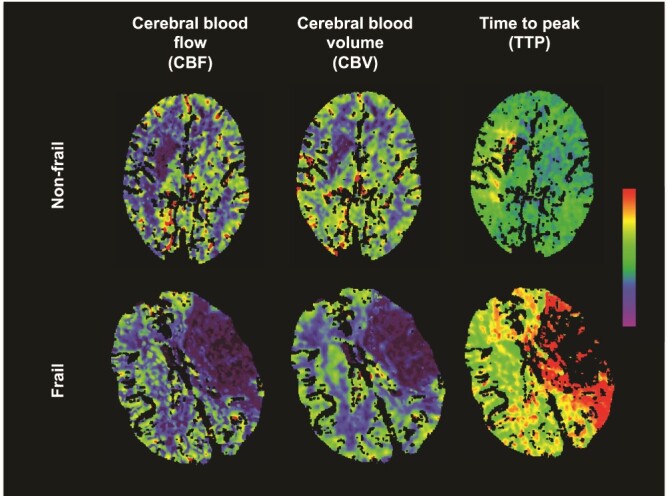
CT Perfusion findings in an individual without frailty (top row) and an individual with frailty (bottom row) presenting at the same time since stroke onset (also of similar age). Both individuals had MCA occlusion. The non-frail individual has a large penumbral fraction as demonstrated by the large mismatch between the CBF and cerebral blood volume (CBV). In contrast, the individual with frailty has very little salvageable brain (as illustrated by little mismatch between the CBF and CBV). A FI cut-off of 0.24 was used between frailty and non-frailty [18].

On univariable analysis, a higher FI was associated with a reduced penumbral fraction (*r_s_* = −0.36, *P* < 0.01). In contrast, there was no relationship between the penumbral fraction and age (*r_s_* = −0.06, *P* = 0.67) or mRS (*r_s_* = 0.17, *P* = 0.23). CTP images of a frail and non-frail individual with middle cerebral artery (MCA) occlusion, presenting at the same time since stroke onset, are shown in Figure 1.

In a dichotomized analysis, individuals with frailty had a smaller median penumbral fraction (0.45, inter-quartile range (IQR) 0.40–0.46) than those without frailty (0.81, IQR 0.70–0.89) (*P* < 0.001).

After adjustment for age, sex, onset-to-CT interval, collateral score and presence of vascular comorbidities, a higher FI remained associated with a reduced penumbral fraction in the acute ischemic lesion (beta = −1.16, *P* < 0.001, using the optimised model shown in Table 3). Consequently, every 0.1 increase in FI was associated with a decrease in penumbral fraction by 0.12.

**Table 3:** Regression showing associations between penumbral fraction and clinical characteristics.

	Beta (95% CI)	Significance
FI	−1.16 (−1.67, −0.66)	*P* < 0.001
Collateral score	0.08 (0.03, 0.14)	*P* < 0.01
Fazeka score	−0.01 (−0.06, 0.05)	*P* = 0.80
Age	0.00 (0.00, 0.01)	*P* = 0.15
Onset-to-imaging interval	0.00 (0.00, 0.00)	*P* = 0.15

CRP was not initially included in the model due to its co-linearity with the FI. For confirmation, inclusion of CRP in the above model had little impact on the results: CRP had no independent effect (beta: 0.00, 95% confidence interval (CI) 0.00, 0.00; *P* = 0.32) and the effect size of FI was only slightly reduced (beta: 1.07, 95% CI −1.61, −0.52; *P* < 0.001). No other effect sizes were affected. Consistent with this, there was no significant mediating effect of CRP on the relationship between FI and penumbral fraction (z-score −1.20, *P* = 0.23).

### Neurological outcomes

On univariable analysis, there was a positive association between FI and presentation NIHSS (*r_s_* = 0.31, *P* = 0.02) and 24-h NIHSS (*r_s_* = 0.36, *P* < 0.01).

There was also a negative association between FI and the proportional improvement in NIHSS within 24-h following thrombolysis (*r_s_* = −0.31, *P* = 0.02), an association which remained significant after adjustment for age, collateral status and time since stroke onset (Table 4). Consequently, every 0.1 increase in the FI attenuated the proportional NIHSS improvement following thrombolysis by 0.2.

**Table 4:** Regression showing associations between proportional improvement in NIHSS and clinical characteristics.

	Beta (95% CI)	Significance
FI	−2.00 (−3.33, −0.66)	*P* < 0.01
Collateral score	−0.13 (−0.28, 0.01)	*P* = 0.08
Fazeka score	0.05 (−0.09, 0.19)	*P* = 0.48
Age	0.00 (−0.01, 0.01)	*P* = 0.92
Onset-to-imaging interval	0.00 (0.00, 0.00)	*P* = 0.10

As with penumbral fraction, inclusion of CRP in the model did not affect the results markedly: CRP had no independent effect (beta: 0.00, 95% CI 0.00, 0.01; *P* = 0.49) and the negative association between FI and proportional improvement in NIHSS was more marked (beta −2.18, 95% CI −3.62, −0.74; *P* < 0.01). There was no effect on the effect sizes of the other covariables. Consistent with this, there was no significant mediating effect of CRP on the relationship between FI and proportional improvement in NIHSS (z-score 0.77, *P* = 0.44).

Using a dichotomized approach to frailty, non-frail individuals showed a median proportional NIHSS improvement of 0.49 (IQR 0.09–0.83), whilst frail individuals showed only a median proportional NIHSS improvement of 0.04 (IQR −0.05– 0.38) (*P* = 0.04).

In contrast to FI, mRS showed only a positive association with presentation NIHSS (*r_s_* = 0.38, *P* < 0.01), but not with 24-h NIHSS (*r_s_* = 0.21, *P* = 0.13) or proportional change in NIHSS 24-h after reperfusion therapy (*r_s_* = −0.05, *P* = 0.72).

### Inflammatory markers

The median CRP at presentation was 3.7 mg/L (IQR 0.3–11.2 mg/L). FI was associated with levels of CRP on admission (*r_s_* = 0.38, *P* < 0.01), but not NLR (*r_s_* = 0.11, *P* = 0.43). CRP was inversely associated with penumbral fraction on univariable analysis (*r_s_* = −0.30, *P* = 0.02), but not with proportional improvement in NIHSS (*r_s_* = −0.14, *P* = 0.32).

## Discussion

Our results demonstrate an independent association between pre-stroke frailty and reduced penumbra in hyperacute ischemic stroke. This finding may help explain the attenuated benefit from reperfusion therapies amongst frailer individuals observed in our cohort and previous studies [6, 7].

Understanding the mechanisms underpinning the relationship between frailty and the response to stroke reperfusion therapies is of vital importance given the prevalence of frailty globally and amongst individuals presenting with stroke [3]. Shifting demographics are likely to exacerbate this challenge as ageing populations further increase the global burden of frailty.

Previous research reported chronological age is negatively associated with penumbral volumes, though not core infarct volumes, in acute stroke [14]. Furthermore, older individuals are reported to have attenuated benefit from mechanical thrombectomy compared to younger individuals, but without increased rates of complications [23]. However, it is likely that chronological age in these studies may act as a surrogate marker of frailty (the prevalence of which increases with age) and its associated physiological changes. Our data is the first to consider age and frailty separately as related, but distinct, covariables in relation to penumbra. Our results indicate that age and frailty showed only a moderate association on univariable analysis, but that age had a negligible effect on penumbra after adjustment for frailty. Such findings may have important implications for case selection for acute reperfusion therapies, supporting that we should be assessing frailty and not excluding individuals from reperfusion therapies based on age alone.

Although the sample size was limited, the lack of an association between mRS and cerebral perfusion parameters and neurological outcomes has important implications for eligibility criteria for research studies. Hyperacute research studies often base inclusion on pre-morbid mRS (frequently 0–1 or 0–2), and our results suggest that frailty status may provide a superior, if not complementary, prognostic marker for study eligibility. The moderate association between FI and mRS in our study is consistent with previous work [24], and indicates they represent related but distinct phenomena. It is plausible that the pathophysiological processes in frailty play more of a role in the evolution of the hyperacute ischemic stroke than the functional/disability status captured by mRS.

The association between frailty, reduced penumbra and CRP (and the lack of mediation by the latter) raises the possibility of inflammation being the mechanism through which frailty affects penumbra, or whether inflammation simply reflects comorbidities associated with higher frailty burden, where frailty is recognised as a pro-inflammatory state [25]. In this regard, vascular risk factors and systemic inflammation associated with frailty may ‘prime’ the brain for injury through association with chronic small vessel disease and by promoting neuroinflammation [10, 26, 27]. Further research is essential to elucidate these mechanisms and whether they may represent targets for therapeutic exploitation.

Our study is limited by its sample size and validation in a larger prospective study would be advantageous: although the marked difference between penumbral fraction between frail and non-frail dichotomized groups means that only a small sample is required to compare the two groups (with a conservative post-hoc sample size calculation showing that only eight participants would be required per group), the spectrum of frailty means that a continuous measure may be more advantageous for accommodating the nuance of clinical practice. Furthermore, evaluation of how frailty relates to final infarct volumes using MRI (incorporating quantification of leukoaraiosis burden), as well as the rate of complications from thrombolysis, would also be useful in further delineating the pathophysiological relationships and guiding clinical decision-making. Linked to this, although there was no overt infection at the time of presentation (a finding supported by the distribution of CRP within the normal range), future work would benefit from considering the early trajectory of inflammatory markers to consider any impact from early infection, as well as consideration of more sensitive markers of inflammation (such as high-sensitivity CRP, which is associated with background atheroinflammation [27]). Longer-term evaluation of functional recovery, such as assessment of mRS at 90 days, would be complementary to measures of neurological recovery in order to guide clinical prognostication and decision-making.

Finally, an important consideration for future research and translation to clinical practice will be the frailty assessment tool used. Although the FI provided an effective retrospective measure of frailty in this study, it is arguably too burdensome to administer in a real-time hyperacute stroke setting [28]. More simple measures, such as the Clinical Frailty Scale, are at least as good at predicting outcomes as more complex frailty measures and future work should evaluate their use as a more practical alternative in time-pressured clinical practice [29].

Our finding that individuals with frailty have reduced salvageable penumbra—an association that is strengthened after adjustment for age, collateral status, small vessel disease burden and time from stroke onset—helps elucidate the mechanisms underlying the treatment-attenuating effects of frailty on hyperacute stroke reperfusion therapies. Further research investigating the links between frailty, systemic inflammation, neuroinflammation and blood–brain barrier integrity may yield further mechanistic information to guide clinical management and opportunities for therapeutic exploitation.

## Research Data Transparency and Availability:

The corresponding author had full access to all the data in the study and takes responsibility for its integrity and the data analysis. The data supporting the findings of this study are available from the corresponding author upon reasonable request. Reporting followed STROBE guidelines.

## References

[1] Clegg A , Young J, Iliffe S et al. Frailty in elderly people. Lancet (London, England). 2013;381:752–62. 23395245 10.1016/S0140-6736(12)62167-9 PMC4098658

[2] Hoogendijk EO , Afilalo J, Ensrud KE et al. Frailty: Implications for clinical practice and public health. Lancet (London, England). 2019;394:1365–75. 31609228 10.1016/S0140-6736(19)31786-6

[3] Burton JK , Stewart J, Blair M et al. Prevalence and implications of frailty in acute stroke: Systematic review & meta-analysis. Age Ageing. 2022;51:afac06. 10.1093/ageing/afac064 PMC9037368 35352795

[4] Evans NR , Todd OM, Minhas JS et al. Frailty and cerebrovascular disease: Concepts and clinical implications for stroke medicine. Int J Stroke. 2021;17:251–9. 34282986 10.1177/17474930211034331 PMC8864332

[5] Munthe-Kaas R , Aam S, Saltvedt I et al. Is frailty index a better predictor than pre-stroke modified Rankin scale for neurocognitive outcomes 3-months post-stroke? BMC Geriatr. 2022;22:139. 35183106 10.1186/s12877-022-02840-y PMC8857811

[6] Evans NR , Wall J, To B et al. Clinical frailty independently predicts early mortality after ischaemic stroke. Age Ageing. 2020;49:588–91. 31951248 10.1093/ageing/afaa004

[7] Joyce N , Atkinson T, Mc Guire K et al. Frailty and stroke thrombectomy outcomes-an observational cohort study. Age Ageing. 2022;51:afab260. 10.1093/ageing/afab260 35150584

[8] Zhang L , Zeng X, He F et al. Inflammatory biomarkers of frailty: A review. Exp Gerontol. 2023;179:112253. 37429425 10.1016/j.exger.2023.112253

[9] Varatharaj A , Galea I. The blood-brain barrier in systemic inflammation. Brain Behav Immun. 2017;60:1–12. 26995317 10.1016/j.bbi.2016.03.010

[10] Evans NR , Tarkin JM, Walsh J et al. Carotid Atheroinflammation is associated with cerebral small vessel disease severity. Front Neurol. 2021;12:690935. 10.3389/fneur.2021.690935 PMC8438317 34531813

[11] Epstein SE , Lassance-Soares RM, Faber JE et al. Effects of aging on the collateral circulation, and therapeutic implications. Circulation. 2012;125:3211–9. 22733335 10.1161/CIRCULATIONAHA.111.079038

[12] Cipolli GC , Ribeiro IC, Yasuda CL et al. Frailty and brain changes in older adults without cognitive impairment: A scoping review. Arch Gerontol Geriatr. 2024;123:105395. 38492289 10.1016/j.archger.2024.105395

[13] Taylor-Rowan M , Hafdi M, Drozdowska B et al. Physical and brain frailty in ischaemic stroke or TIA: Shared occurrence and outcomes. Cohort Study Eur Stroke J. 2023;8:1011–20. 37421136 10.1177/23969873231186480 PMC10683729

[14] Agarwal S , Scoffings DJ, Jones PS et al. Interaction of age with the ischaemic penumbra, leptomeningeal collateral circulation and haemodynamic variables in acute stroke: A pilot study. J Neurol Neurosurg Psychiatry. 2013;84:271–6. 23178505 10.1136/jnnp-2012-303258

[15] Taylor-Rowan M , Cuthbertson G, Keir R et al. The prevalence of frailty among acute stroke patients, and evaluation of method of assessment. Clin Rehabil. 2019;33:1688–96. 30971115 10.1177/0269215519841417

[16] Gupta K , Williams E, Warburton EA et al. Pre-stroke frailty and outcomes following percutaneous endoscopic gastrostomy tube insertion. Healthcare (Basel). 2024;12:1557. 10.3390/healthcare12161557 PMC11353361 39201117

[17] Theou O , Haviva C, Wallace L et al. How to construct a frailty index from an existing dataset in 10 steps. Age Ageing. 2023;52:afad221. 10.1093/ageing/afad221 PMC10733590 38124255

[18] Rockwood K , Andrew M, Mitnitski A. A comparison of two approaches to measuring frailty in elderly people. J Gerontol A Biol Sci Med Sci. 2007;62:738–43. 17634321 10.1093/gerona/62.7.738

[19] Maas MB , Lev MH, Ay H et al. Collateral vessels on CT angiography predict outcome in acute ischemic stroke. Stroke. 2009;40:3001–5. 19590055 10.1161/STROKEAHA.109.552513 PMC2754152

[20] Rudilosso S , San Román L, Blasco J et al. Evaluation of white matter hypodensities on computed tomography in stroke patients using the Fazekas score. Clin Imaging. 2017;46:24–7. 28688243 10.1016/j.clinimag.2017.06.011

[21] Akaike H . Information theory and an extension of the maximum likelihood principle. In: Petrov BN, Csaki BF (eds.), *Second International Symposium on Information Theory*, 1973. Budapest: Academiai Kiado, pp. 267–81.

[22] Bozdogan H . Model selection and Akaike's information criterion (AIC): The general theory and its analytical extensions. Psychometrika. 1987;52:345–70.

[23] McDonough RV , Ospel JM, Campbell BCV et al. Functional outcomes of patients ≥85 years with acute ischemic stroke following EVT: A HERMES substudy. Stroke. 2022;53:2220–6. 35703094 10.1161/STROKEAHA.121.037770

[24] Fearon P , McArthur KS, Garrity K et al. Prestroke modified Rankin stroke scale has moderate interobserver reliability and validity in an acute stroke setting. Stroke. 2012;43:3184–8. 23150650 10.1161/STROKEAHA.112.670422

[25] van Epps P , Oswald D, Higgins PA et al. Frailty has a stronger association with inflammation than age in older veterans. Immunity Ageing. 2016;13:27. 27777599 10.1186/s12979-016-0082-z PMC5069820

[26] Drake C , Boutin H, Jones MS et al. Brain inflammation is induced by co-morbidities and risk factors for stroke. Brain Behav Immun. 2011;25:1113–22. 21356305 10.1016/j.bbi.2011.02.008 PMC3145158

[27] Evans NR , Tarkin JM, Chowdhury MM et al. Dual-tracer positron-emission tomography for identification of culprit carotid plaques and pathophysiology In vivo. Circ Cardiovasc Imaging. 2020;13:e009539. 32164454 10.1161/CIRCIMAGING.119.009539

[28] Evans NR , Fearon P, Beishon L et al. The importance of frailty in stroke and how to measure it. Stroke. 2024;0(online ahead of print). 10.1161/STROKEAHA.124.048424 39234696

[29] Boucher EL , Gan JM, Rothwell PM et al. Prevalence and outcomes of frailty in unplanned hospital admissions: A systematic review and meta-analysis of hospital-wide and general (internal) medicine cohorts. EClinicalMedicine. 2023;59:101947. 37138587 10.1016/j.eclinm.2023.101947 PMC10149337

